# Response to “Comment on ‘An Informatics Approach to Evaluating Combined Chemical Exposures from Consumer Products: A Case Study of Asthma-Associated Chemicals and Potential Endocrine Disruptors’”

**DOI:** 10.1289/EHP778

**Published:** 2016-09-01

**Authors:** Henry A. Gabb, Catherine Blake

**Affiliations:** Graduate School of Library and Information Science, University of Illinois at Urbana–Champaign, Champaign, Illinois, USA

Neither informatics (e.g., Goldsmith et al. 2014) nor gas chromatography–mass spectrometry (GC/MS) (e.g., Steinemann et al. 2011, Dodson et al. 2012, Steinemann 2015) can provide a complete picture of the ingredients in consumer products. Our article demonstrates the complementary nature of these approaches. The larger product sample of the informatics approach gives greater coverage of the formulation space of consumer products. Consequently, it detected the target chemicals in product categories that were missed by GC/MS. However, the informatics approach is limited by the incompleteness of product labels, particularly with respect to fragrance and flavor mixtures. GC/MS can detect chemicals that are not disclosed in product labels and chemicals that are not even part of the product formulation (e.g., chemicals leached from product packaging or degradation byproducts). However, the small sample sizes, typically only a handful of products in a given category, do not reflect the diversity of product formulations.

As with consumers themselves, our informatics approach only has access to information that is available on a product label, but it can scale to a larger set of products. Time and cost limit the number of products and the specific chemicals that can be detected using GC/MS. We did not include fragrances in the initial analysis because the generic term “fragrance” lacks the specificity necessary to establish if a product actually contains one of the target chemicals identified by Dodson et al. (2012). Steinemann et al. (2011) and Steinemann (2015) show the broad range of fragrance chemicals in consumer products. As mentioned in our article, generic fragrance is the second most common ingredient in our product sample after water. Treating generic fragrance as a target chemical (even though it is a mixture) would have increased the number of products by 4,043 (10% of the total sample of 38,975 products).

Steinemann’s point about overreliance on publicly disclosed ingredient information is well taken. In the United States, labeling of consumer products is governed by many overlapping statutes, the Consumer Product Safety Act and the Fair Packaging and Labeling Act (FPLA) among them. However, the FPLA definition of a consumer product (§503.2, §503.5) encompasses nearly all the product categories in our analysis (GPO 1971). Only pet care products, which made up 1.6% of our sample, are excluded from this definition (FPLA §503.5.d.7) (GPO 1971). Although manufacturers are required by the FPLA (§1454.c.3.B) to list ingredients in order of decreasing predominance, they are not required to divulge trade secrets (GPO 2009). The fragrance and flavor mixtures in many consumer products are often treated as trade secrets, so they are simply listed generically as “fragrance” or “flavor” on the product label.

Another key result of our analysis is the degree to which chemical synonymy (i.e., different names for the same chemical) further obfuscates product labels, especially from the consumer standpoint. A consumer cannot be reasonably expected to recognize all names for a chemical ingredient. Our article presented possible scenarios illustrating how chemical synonymy and the generic “fragrance” designation (or sometimes even its absence) on product labels can mislead consumers or give them a false sense of security.

Ultimately, combining informatics and GC/MS will help to ensure that we have good coverage with respect to both the number of products and the number of chemicals that can be detected. The informatics approach could also be used by GC/MS researchers to prioritize which product groups to analyze. We encourage further debate on the shortcomings of consumer product labels and labeling regulations so that consumers and informatics approaches can be better informed about the ingredients in the products we buy.
